# Explore on screening COX-2 inhibitors from the essential oil of *Solanum lyratum* Thunb. By molecular docking and molecular dynamics simulation

**DOI:** 10.1016/j.heliyon.2024.e37652

**Published:** 2024-09-07

**Authors:** Hanyang Xiao, Yan Gui, Xianfei Li, Wen Dai, Chuanhua Feng, Gang Li, Jiangnan Luo

**Affiliations:** a Department of Pharmacy, 908th Hospital of the PLA Joint Logistics Support Force, Nanchang, 330002, Jiangxi, PR China; b Department of Pharmacy, the First Affiliated Hospital, Jiangxi Medical College, Nanchang University, Nanchang, 330006, Jiangxi, PR China; c Institute of Traditional Chinese Medicine Health Industry, China Academy of Chinese Medical Sciences, Nanchang, 330115, Jiangxi, PR China

**Keywords:** Essential oil, *Solanum lyratum* Thunb., Molecular docking, Molecular dynamics simulation, COX-2 enzyme

## Abstract

This study aimed to investigate *Solanum lyratum* Thunb. with respect to the potential ingredients with anti-inflammatory activity from its essential oil by *silico* study. To this regard, the essential oil of *Solanum lyratum* Thunb. was extracted by hydrodistillation. 25 compounds were identified by GC-MS. Using virtual screening, molecular docking and molecular dynamics simulation of the 25 identified compounds, the ones showing anti-inflammatory activity on COX-2 were identified. According to the drug-like principle and the prediction of ADEMT properties, the six compounds of Spathulenol, Cedrol, Juniper camphor, Santalol, Nootkatone and 7,9-Di-tert-butyl-1-oxaspiro[4.5]deca-6,9-diene-2,8-dione were identified and then studied for molecular docking, and based on which the top two compounds of binding free energy were studied by the molecular dynamics simulation. The molecular docking data indicated that the binding free energies of Spathulenol, Cedrol, Juniper camphor, Santalol, Nootkatone and 7,9-Di-tert-butyl-1-oxaspiro[4.5]deca-6,9-diene-2,8-dione to COX-2 protein were −5.65, −7.19, −6.35, −4.94, −5.82 and −5.14 kcal/mol, respectively. The findings showed the steady interactions of hydrogen bonds and hydrophobic bonds between both the top two compounds of binding free energy and the active site residues of COX-2 (4M11) throughout the simulation via hydrogen bonds and hydrophobic bonds. The very study shall be supportive for in vitro and in vivo studies in developing drug products using the lead bioactive ingredients for anti-inflammatory in the future.

## 1 Introduction

Cyclooxygenase (COX) is an enzyme playing critical part in generating prostaglandins and leukotrienes from arachidonic acid. Selective inhibition of COX can be used to treat many diseases, such as antipyretic, analgesic and anti-inflammatory [[1], [2], [3], [4], [5]]. As an inducible enzyme, the expression of COX is rarely found in normal cells, but high expression levels are always found in the course of inflammation. It mediates the expression of pro-inflammatory mediators and cytokines, thereby playing a vital part in the pathological process of inflammation [[6], [7], [8]]. Therefore, developing medicines that can effectively inhibit COX-2 will be of great significance for treating inflammatory diseases.

The search for effective compounds from natural drugs has gained more popularity in recent years. Solanum lyratum Thunb., which belongs to the Solanaceae family, has been utilized as a Chinese medicinal material more than 2000 years. Studies have confirmed that *Solanum lyratum* Thunb. contains rich chemical components, such as flavonoids, terpenoids, organic acids, saponins, essential oils, and has a extensive range of pharmacological effects, including immune regulation, antibacterial, anti-inflammatory, anticancer, antioxidant and so on, and is clinically used for treating rheumatoid arthritis, hepatitis, cancer, malaria, cold and other diseases [[9], [10], [11], [12], [13], [14]]. The essential oil of *Solanum lyratum* Thunb. is a very important class of compounds, mainly including monoterpenes, sesquiterpenes and their oxides. Modern studies have found that essential oil usually has better anti-inflammatory and antioxidant effects. However, the actually effective chemical components contributing to the better anti-inflammatory effects of the essential oil remain unknown. Herein, using virtual screening technology, the interactions between identified chemical components of the essential oil from *Solanum lyratum* Thunb. and COX-2 were simulated and calculated. Screening anti-inflammatory lead compounds to improve the efficiency of new drug discovery [[15], [16], [17], [18]].

Herein, the essential oil of *Solanum lyratum* Thunb. was prepared to identify the chemical components with anti-inflammatory effects and also to study the underlying mechanism. The essential oil of *Solanum lyratum* Thunb. was prepared by hydrodistillation, then the compounds in the essential oil were analyzed and identified using GC-MS technique, and the chemochemical characteristics of the identified compounds were investigated in *silico* study. Finally, the compounds that meet the principle of drug-like and ADMET characteristics were used for molecular docking and molecular dynamics simulation to screen the lead compounds that can effectively inhibit COX-2, providing important theoretical basis for in *vivo* and in *vitro* experimental studies in the future.

## 2 Materials and methods

### 2.1 Plant materials

The grass of *Solanum lyratum* Thunb. was sourced from Zhangshu, (Jiangxi province, China) in June 2023, and has been identified by a professor from Jiangxi University of Chinese Medicine in accordance with the Pharmacopoeia of the People's Republic of China. The voucher specimen (XF20220306002) was deposited also at the Jiangxi University of Chinese Medicine. After being completely dried, the collected grass of *Solanum lyratum* Thunb. were ground into powders, and then the powders were sifted using 40 mesh sieve, and finally the sieved materials were packaged in well-closed PE bags before further use.

### 2.2 Chemicals and reagent

n-Hexane (HPLC grade, Yonghua Chemical Co., LTD., batch number: 20200326); anhydrous sodium sulfate (Analytical pure, Xilong Science Co., LTD., batch number: 1905082); helium (Nanchang jiangzhu Industrial Co., LTD., purity greater than 99.999 %); all other reagents sourced from Damao Chemical Reagent Factory (Tianjin, China). The de-ionised water was produced in-house using a Hitech water system (Shanghai, China).

### 2.3 Essential oil Extractions

The powder (100g) of the dried grass of *Solanum lyratum* Thunb. was subjected to 4-h hydrodistillation using a clevenger apparatus to prepare the essential oil, afterward, the resultant was dried with addition of anhydrous sodium sulfate, then sealed in dark vials, and stored at 4 °C before further use.

### 2.4 Gas chromatography-mass spectrometry (GC-MS)

The sample were analyzed by Agilent 7890A GC system and 5975C MS system using a column packed with HP-5 MS (5 % phenylmethylpolysiloxane, 30 m × 0.25 mm × 0.25 μm; Agilent, America). For the test conditions, the oven temperature was initially maintained at 50 °C for 2 min and then ramped to 250 °C by 4 °C/min, and finally maintained at 250 °C for 15 min; the injector port temperature was 250 °C; the transfer interface and source temperature was 250 °C; the flow rate of carrier gas (He) was 1 mL/min; the electron ionization source was 70 ev; the scanning was performed at a rate was 0.5 s (cycle time: 0.2 s) within50–400 amu. The various compounds were identified through a comparison of between the mass spectra of detected compounds and the NIST Mass Spectral library (NIST 08). The relative contents (%) of the volatile components were calculated by the area normalization method.

### 2.5 Compounds database

The compounds of the essential oil from the dry above-ground plant of *Solanum lyratum* Thunb. were prepared with the 3D SDF files downloaded from the PubChem database [19,20].

### 2.6 Drug-likeness properties

The predictions of drug-like properties for the identified compounds were performed with the SwissADME online database. By entering the SMILES notations sourced from the PubChem database for the identified compounds into SwissADME, the drug-like properties of important molecular properties were calculated. Then in accordance with the criteria followed in drug design, screening with respect to drug likeliness was performed for the compounds. The major criteria considered in this study were the Lipinski rule, Veber's Law, molecular weight, number of hydrogen donors, number of hydrogen acceptors, number of rotatable bonds, and so on [[21], [22], [23], [24], [25], [26]].

### 2.7 Bio-activity scores prediction

The calculation of bio-activity scores for the compounds were completed by online tool—Molinspiration. The important parameters include GPCR ligands, protease inhibitors, kinase inhibitors, ion channel modulators, nuclear receptors, and enzyme inhibitors. Based on this, the compounds with positive enzyme inhibitor scores were selected for subsequent studies. The CLogP value of a compound is an important parameter to measure its hydrophilicity [27,28]. Herein, the calculation of CLogP values for the selected compounds was completed using the ChemBioDraw Ultra 14.0 software.

### 2.8 Toxicity prediction

As an important parameter for evaluation of drug safety, toxicity is always of high concern. In this study, ProTox-II webserver was searched for the toxicity of the selected compounds, and then the compounds were classified regarding their toxicity according to the LD50 data [29].

### 2.9 Protein preparation

The high-resolution (2.45 Å) X-ray crystal structure of the chosen enzyme target of COX-2 protein (PDB ID: 4M11) was sourced from RCSB PDB database. The chosen enzyme targets for molecular docking were prepared by removing hetero molecules and water molecules using Pymol 1.8. The A chain of the target protein was selected for molecular docking study. The prepared proteins were saved as PDBQT format after adding hydrogen atoms and charges [[30], [31], [32]].

### 2.10 Ligand preparation

The selected compounds in the essential oil, which conforms to the drug-like criterions, positive enzyme inhibitor scores and toxicity classes 4 or above, were treated with PMV 1.5.6 and saved as PDBQT for molecular docking.

### 2.11 Molecular docking

Using PMV 1.5.6, the selected compounds were investigated for their molecular interactions to the target protein (COX-2) were studied using PMV 1.5.6. In the process of molecular docking, the ligands and target proteins were defined as flexible and rigid molecules, respectively. Herein, the method adopted was blind docking. The grid boxes covers the entire surface of the proteins to ensure docking with the selected ligands. The docking was performed for 50 runs, and the molecular interactions were visualized with Pymol 1.8. For the molecular dynamics simulation of the ligands, the lowest energy conformation were used as input [33,34].

### 2.12 Molecular dynamics simulation

The molecular dynamics simulation can elucidate the conformation change of the ligand-receptor complexes under simulation conditions. In this study, the simulation was performed by GROMACS 2020.6 package implemented with CHARMM36 force field for the complexes of the top two compounds of binding free energy (Cedrol and Juniper camphor) with target protein (4M11). First, the topology files of receptor and ligand were prepared respectively, and the topology file of ligand-receptor complex was generated. Then define the unit cell and add solvent and ions to the unit cell to make the whole system neutral; the simulation was carried out after optimizing the system to minimize the system energy and balancing the ligand-receptor complex. After the simulation, the ligand-receptor interaction and ligand dynamics were analyzed [35,36].

## 3 Results

### 3.1 Analysis of chemical composition

**Fig. 1:**
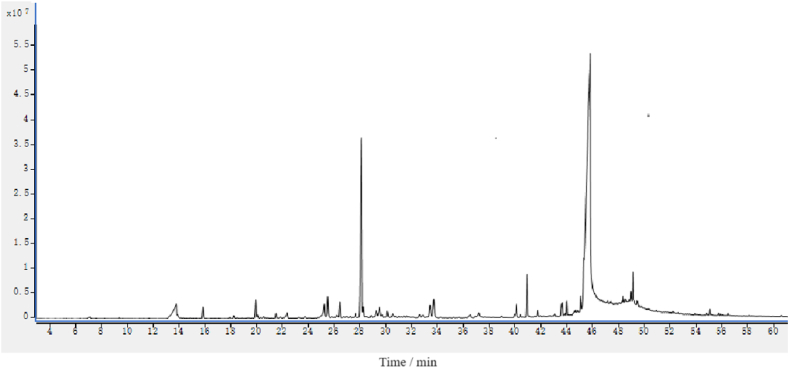
TIC chromatogram of essential oil from *Solanum lyratum* Thunb.

The yield of essential oil from *Solanum lyratum* Thunb. was (0.84 ± 0.25)% (w/w). The TIC chromatogram of the essential oil is presented in Fig. 1, and its chemical composition by HD was determined and provided in Table 1, from which, it is known that there are 25 chemical components were identified from the essential oil by GC-MS, including mainly sesquiterpenes, oxidized sesquiterpenes, monoterpenes and fatty acid.

**Table 1:** Chemical composition of essential oil from *Solanum lyratum* Thunb.

No.	RT	CAS	Name	MF	MW	Content (%)
1	12.366	112-05-0	Nonanoic acid	C9H18O2	158.24	0.51
2	15.68	334-48-5	Decanoic acid	C10H20O2	172.26	5.35
3	17.17	489-39-4	Aromadendrene	C15H24	204.35	0.79
4	19.028	644-30-4	α-Curcumene	C15H22	202.34	1.41
5	19.793	495-61-4	β-Bisabolene	C15H24	204.35	0.56
6	20.251	30021-74- 0	γ-Muurolene	C15H24	204.35	0.74
7	21.45	143-07-7	Lauric acid	C12H24O2	200.32	2.73
8	21.843	6750-60-3	Spathulenol	C15H24O	220.35	0.46
9	22.018	1139-30-6	Caryophyllene oxide	C15H24O	220.35	1.76
10	22.545	77-53-2	Cedrol	C15H26O	222.37	1.52
11	23.369	515-17-3	γ-Selinene	C15H24	204.35	0.37
12	23.89	51317-08- 9	Eudesmol	C15H26O	222.37	0.78
13	24.017	473-04-1	Juniper camphor	C15H26O	222.37	1.64
14	25.529	11031-45- 1	Santalol	C15H24O	220.35	0.61
15	25.951	2298-07-9	4-Bromo-1-naphthylamine	C10H8BrN	222.08	3.72
16	27.292	544-63-8	Myristic acid	C14H28O2	228.37	0.74
17	28.865	4674-50-4	Nootkatone	C15H22O	218.33	16.87
18	30.686	502-69-2	Fitone	C18H36O	268.48	2.84
19	31.83	84-64-0	Cyclohexyl butyl phthalate	C18H24O4	304.38	0.37
20	34.711	82304-66- 3	7,9-Di-tert-butyl-1-oxaspiro[4.5]deca-6,9-diene-2,8-dione	C17H24O3	276.37	1.37
21	35.272	112-39-0	Methyl hexadecanoate	C17H34O2	270.45	0.68
22	37.323	89-18-9	Phthalic acid, butyl 8-methylnonyl ester	C22H34O4	362.5	0.51
23	47.492	60-33-3	Linoleic acid	C18H32O2	280.45	48.2
24	49.051	2937-53-3	Cysteaminesulfonic acid	C2H7NO3S2	157.21	0.4
25	56.847	96168-15- 9	4,8,12,16-Tetramethylheptadecan-4-olide	C21H40O2	324.54	0.34

### 3.2 ADMET/drug-likeness properties and bio-activity score prediction of selected ligands

Using Swiss ADME, the prediction of drug-likeness properties was performed for the 25 identified chemical compounds. The method was able to remove compounds without obvious drug-likeness properties. The rules should be followed during the drug-likeness properties screening process: Lipinski rule, Veber rule, MW < 500, number of hydrogen donors (≤5), hydrogen acceptors (≤10) and rotatable bonds (≤10), as well as total polar surface area ≤140 Å^2^. The calculation of CLogP values for the compounds were performed with ChemBioDraw Ultra 14.0 software, and it is negatively correlated with the water permeability. The compounds with a CLogP value of <5 were selected for subsequent analyses. Therefore, according to the results of ADME and CLogP value, 10 compounds out of 25 compounds conform to the drug-likeness criterions. The physical-chemical properties are presented in Table 2, and the ADME properties are presented in Table 3.

**Table 2:** Physical–chemical properties of 10 compounds conform to the drug-likeness criterions.

No.	Name	MV	HBA	HBD	RB	TPSA	CLogP
1	Nonanoic acid	158.238	2	1	7	37.3	3.509
2	Decanoic acid	172.265	2	1	8	37.3	4.038
3	Spathulenol	220.35	1	1	0	20.23	4.306
4	Caryophyllene oxide	220.35	1	0	0	12.53	4.743
5	Cedrol	222.366	1	1	0	20.23	4.53
6	Juniper camphor	222.366	1	1	0	20.23	4.7
7	Santalol	220.35	1	1	4	20.23	4.946
8	4-Bromo-1-naphthylamine	222.081	0	1	0	26.02	3.232
9	Nootkatone	218.335	1	0	1	17.07	4.154
10	7,9-Di-tert-butyl-1-oxaspiro[4.5]deca-6,9-diene-2,8-dione	276.371	3	0	2	43.37	2.423

**Table 3:** ADME properties and the drug-likeness of 10 compounds conform to the drug-likeness criterions.

No.	GI	BBB	P-gp substrate	CYP inhibitor					Lipinski	Veber
				CYP1A2	CYP2C19	CYP2C9	CYP2D6	CYP3A4		
1	High	Yes	No	No	No	No	No	No	YES	YES
2	High	Yes	No	No	No	No	No	No	YES	YES
3	High	Yes	No	No	Yes	No	No	No	YES	YES
4	High	Yes	No	No	Yes	Yes	No	No	YES	YES
5	High	Yes	No	No	No	Yes	No	No	YES	YES
6	High	Yes	No	No	No	Yes	No	No	YES	YES
7	High	Yes	No	No	Yes	Yes	No	No	YES	YES
8	High	Yes	No	Yes	Yes	Yes	No	No	YES	YES
9	High	Yes	No	No	Yes	Yes	No	No	YES	YES
10	High	Yes	No	Yes	No	No	No	No	YES	YES

Using the ProTox-II webserver, the prediction of toxicity was performed for the selected compounds. The compounds of LD50 category IV/V/VI, which were not hepatotoxic, carcinogenic, immunotoxic, mutagenic and cytotoxic, were selected for subsequent molecular docking analyses. The results showed that among the 10 compounds, except Caryophyllene oxide and 4-Bromo-1-naphthylamine, the other 8 compounds were less toxic. The toxicity prediction results of the compounds are presented in Table 4.

**Table 4:** The toxicity prediction results of the selected compounds.

No.	Name	Toxicity Class	Hepatotoxicity	Carcinogenicity	Immunotoxicity	Mutagenicity	Cytotoxicity
1	Nonanoic acid	4	Inactive	Inactive	Inactive	Inactive	Inactive
2	Decanoic acid	4	Inactive	Inactive	Inactive	Inactive	Inactive
3	Spathulenol	5	Inactive	Inactive	Inactive	Inactive	Inactive
4	Cedrol	4	Inactive	Inactive	Inactive	Inactive	Inactive
5	Juniper camphor	5	Inactive	Inactive	Inactive	Inactive	Inactive
6	Santalol	5	Inactive	Inactive	Inactive	Inactive	Inactive
7	Nootkatone	6	Inactive	Inactive	Inactive	Inactive	Inactive
8	7,9-Di-tert-butyl-1-oxaspiro[4.5]deca-6,9-diene-2,8-dione	4	Inactive	Inactive	Inactive	Inactive	Inactive

In general, it is assumed that a compound is considered as active, moderately active, and inactive for a bioactivity score of >0, −0.5–0, and < −0.5. In this study, the online website Molinspiration was used to score the biological activities of compounds, and compounds with a positive enzyme inhibitor score were selected for subsequent studies. The results showed that 6 compounds had enzyme inhibitor scores greater than zero, namely Spathulenol, Cedrol, Juniper camphor, Santalol, Nootkatone and 7,9-Di-tert-butyl-1-oxaspiro[4.5]deca-6,9-diene-2,8-dione. Therefore, they were selected for subsequent molecular docking studies. The bio-activity scores of the selected compounds were shown in Table 5.

**Table 5:** Bio-activity scores of the selected compounds.

No.	Name	GPCR ligand	Ion channel modulator	Kinase inhibitor	Nuclear receptor ligand	Protease inhibitor	Enzyme inhibitor
1	Spathulenol	−0.42	−0.28	−0.68	0.28	−0.36	0.06
2	Cedrol	−0.15	0.27	−0.94	0.03	−0.52	0.50
3	Juniper camphor	−0.19	0.18	−0.83	0.51	−0.70	0.29
4	Santalol	−0.05	−0.03	−0.23	−0.05	−0.1	0.01
5	Nootkatone	−0.4	−0.31	−1.73	0.66	−0.58	0.34
6	7,9-Di-tert-butyl-1-oxaspiro[4.5]deca-6,9-diene-2,8-dione	−0.17	−0.26	−0.39	0.04	−0.25	0.15

### 3.3 Molecular docking

Molecular docking is a very effective analysis for the prediction of interactions between ligands and proteins. Herein, PMV.1.5.6 software was used to dock the four screened compounds (Spathulenol, Cedrol, Juniper camphor, Santalol, Nootkatone and 7,9-Di-tert-butyl-1-oxaspiro[4.5]deca-6,9-diene-2,8-dione) with the COX-2 protein, so as to obtain the binding free energy with the target protein COX-2 for each of them. Then the compounds with the lowest binding free energy were selected for the visualization, and the interaction between the ligand and the COX-2 protein was visualized using PyMol 1.8. The molecular docking results showed that the binding free energy of was −5.65, −7.19, −6.35, −4.94, −5.82 and −5.14 kcal/mol, Spathulenol, Cedrol, Juniper camphor, Santalol, Nootkatone and 7,9-Di-tert-butyl-1-oxaspiro[4.5]deca-6,9-diene-2,8-dione with COX-2 protein, respectively, and was was −5.64 kcal/mol for the co-crystalline compound Meloxicam with COX-2 protein, indicating that Spathulenol, Cedrol, Juniper camphor and Nootkatone had a strong affinity with COX-2 protein.

**Fig. 2:**
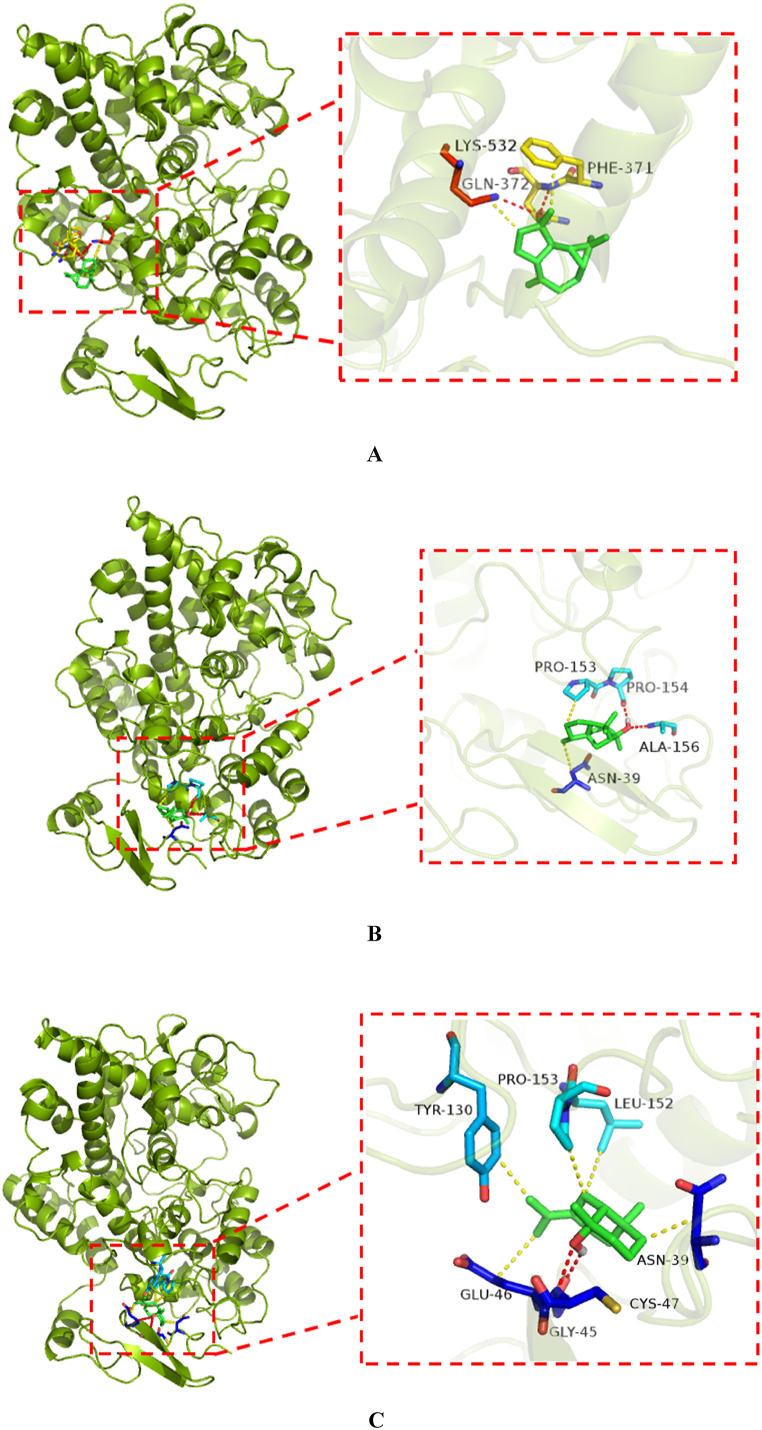

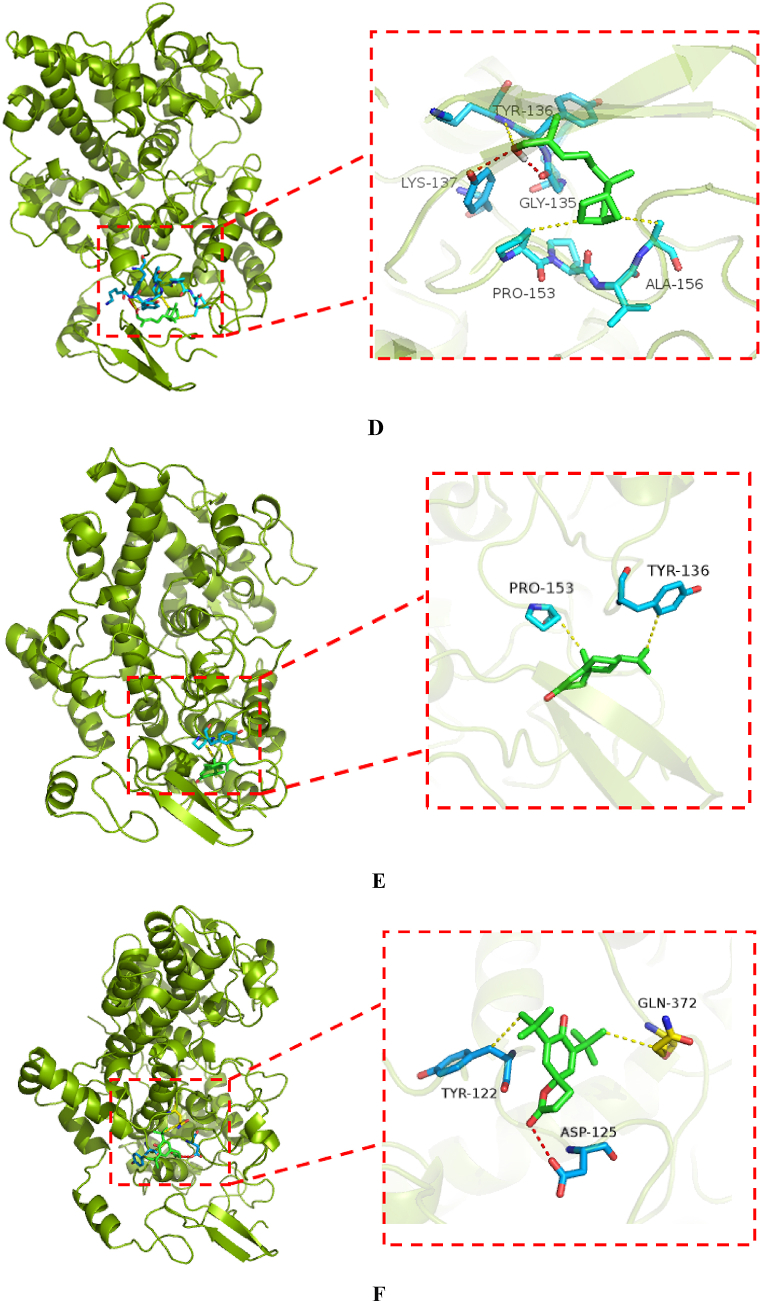
The diagrams for the interaction between ligand and COX-2 protein. The image was made by using PyMol 1.8. (A: Spathulenol, B: Cedrol; C: Juniper camphor, D: Santalol, E: Nootkatone, F: "https://pubchem.ncbi.nlm.nih.gov/compound/545303"7,9-Di-tert-butyl-1-oxaspiro[4.5]deca-6,9-diene-2,8-dione)

The interaction between ligand and protein mainly includes hydrogen bond interactions, hydrophobic bond interactions, Vander Waal interactions and so on. The interaction between the compound and COX-2 protein was shown in Fig. 2. From Fig. 2A–F, it can be seen that Spathulenol (2A) had hydrogen bonding with GLN-372, LYS-532 and had hydrophobic bonding with PHE-371 and LYS-532; Cedrol (2B) had hydrogen bonding with PRO-154 and ALA-156, had hydrophobic bonding with ASN-39 and PRO-153; Juniper camphor (2C) had hydrogen bonding with GLY-45, CYS-47, had hydrophobic bonding with ASN-39, GLU-46 TYR-130, LEU-152 and PRO-153; Santalol (2D) had hydrogen bonding with GLY-135, LYS-137, had hydrophobic bonding with TYR-136, PRO-153 and ALA-156; Nootkatone (2E) had hydrophobic bonding with TYR-136, PRO-153; 7,9-Di-tert-butyl-1-oxaspiro[4.5]deca-6,9-diene-2,8-dione (2F) had hydrogen bonding with ASP-125, had hydrophobic bonding with TYR-122, GLU-372.

### 3.4 Molecular dynamics simulation

**Fig. 3:**
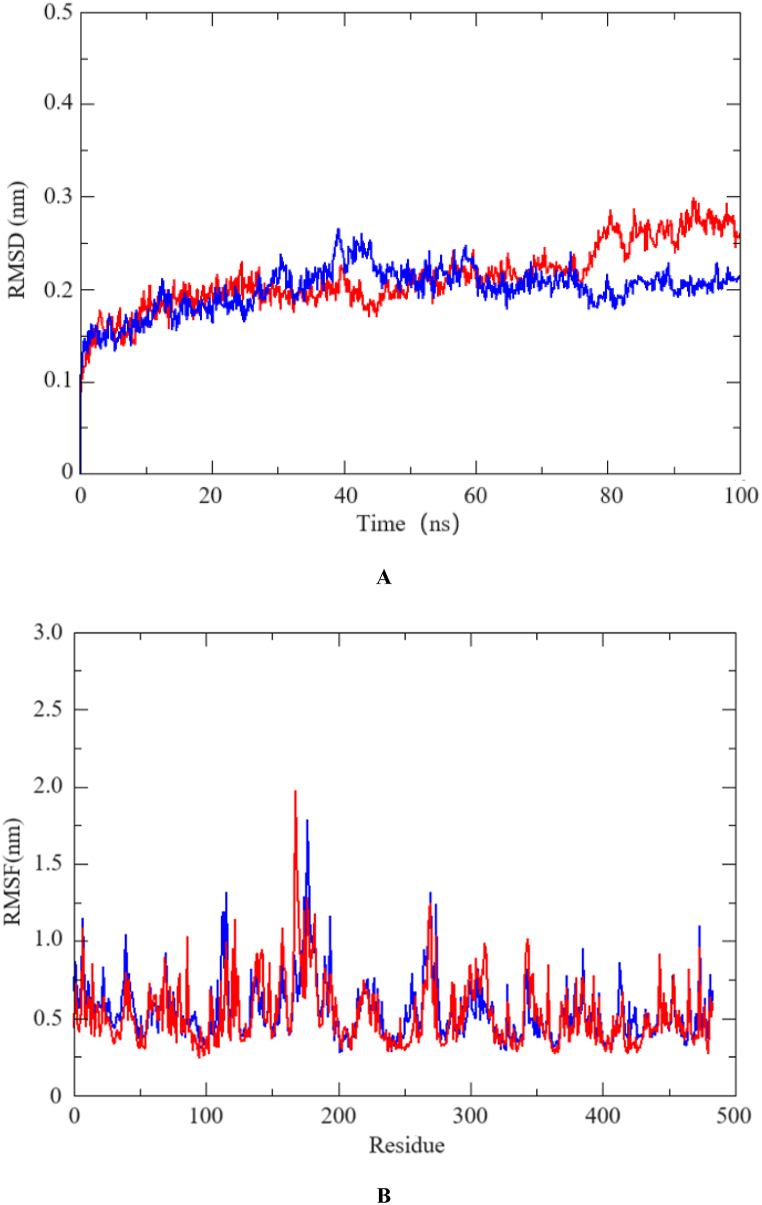
Molecular dynamics simulation trajectory plots (A:RMSD; B:RMSF).

For Cedrol and Juniper camphor, which showed top two binding free energy docked ligand–receptor complexes, Cedrol and Juniper camphor, the molecular dynamics simulations were performed. Fig. 3 showed the RMSD and RMSF between the COX-2 protein (4M11) receptor with Cedrol (Fig. 3A) and Juniper camphor (Fig. 3B), respectively.

#### 3.4.1 Root mean square deviation (RMSD)

The parameter of RMSD represents the stability of conformation for the ligand-receptor complex under the conditions during simulation. The ligand-receptor complex's 100-ns trajectory can be seen in Fig. 3A. As seen in the figure, the molecular dynamics simulation does not result in large variations of the complex, and the average RMSD of all ligand-receptor complexes was around 0.2 nm. The stability of the ligand-receptor complexes was demonstrated by the absence of significant deviations on the trajectory diagram within 100 ns.

#### 3.4.2 Root mean square fluctuation (RMSF)

Molecular dynamics simulations reveal the flexibility of proteins through RMSF. The protein's flexibility decreases after the drug binds to it, which leads to its role as a stabilizing agent. Fig. 3B shows the RMSF of the 500 residues trajectory of the ligand-receptor complex. Fig. 3B shows that the entire sequence segment of the protein has a low RMSF value. The fluctuation of most amino acid residues fluctuate were within 0.25–1.25 nm (0.5–2.0 nm of fluctuation at 160–190 locations), this indicating that the compound can form a stable complex with the key amino acids of the protein, which is conducive to the interaction of ligand and receptor.

## 4 Conclusion

The present *silico* study aims to screen out phytochemical as lead compounds from the essential oil of *Solanum lyratum* Thunb., which may be potential potent ingredients in inhibiting COX-2 protein (4M11). In this study, 25 phytochemical compounds were identified from the essential oil of *Solanum lyratum* Thunb. by GC-MS. For these compounds, the drug-like properties were predicted and then analyzed in accordance with Lipinski's rules, Veber's rules, toxicity rules and bioactivity rules for oral drugs. As a result, Spathulenol, Cedrol, Juniper camphor, Santalol, Nootkatone and 7,9-Di-tert-butyl-1-oxaspiro[4.5]deca-6,9-diene-2,8-dione were identified as potential lead phytochemical compounds for treatment of inflammatory diseases; and these compounds were also proved to show strong binding affinities with 4M11 by the molecular docking analyses. Furthermore, the docked complexes of these compounds were demonstrated to be stable under the conditions during MD simulation. The present work lays down the basis for these theoretical studies and offers insight into the understanding of in vitro and in vivo tests, in the future.

## Funding

This work was supported by the Science and technology project of Jiangxi Provincial Administration of Traditional Chinese Medicine (No.20204816; No.2019A155).

## Data availability statement

The data presented in this study are available on request from the corresponding author.

## Disclosure statement

The authors declare that they have no conflict of interest.

## CRediT authorship contribution statement

**Hanyang Xiao:** Software, Funding acquisition. **Yan Gui:** Writing – original draft. **Xianfei Li:** Methodology. **Wen Dai:** Data curation. **Chuanhua Feng:** Methodology. **Gang Li:** Formal analysis. **Jiangnan Luo:** Conceptualization.

## Declaration of competing interest

The authors declare that they have no known competing financial interests or personal relationships that could have appeared to influence the work reported in this paper.
